# Durability of treatment effects following internet‐delivered cognitive behavioural therapy for depression and anxiety delivered within a routine care setting

**DOI:** 10.1002/cpp.2743

**Published:** 2022-04-26

**Authors:** Jorge E. Palacios, Angel Enrique, Olwyn Mooney, Simon Farrell, Caroline Earley, Daniel Duffy, Nora Eilert, Siobhan Harty, Ladislav Timulak, Derek Richards

**Affiliations:** ^1^ SilverCloud Science SilverCloud Health Dublin Ireland; ^2^ E‐mental Health Research Group, School of Psychology, Aras an Phiarsaigh Trinity College Dublin Dublin Ireland

**Keywords:** anxiety, depression, iCBT, relapse, remission

## Abstract

**Objective:**

To investigate post‐treatment relapse and remission rates 3, 6 and 9 months after completion of an acute phase of a clinician‐supported internet‐delivered cognitive‐behavioural therapy (iCBT) for anxiety and depressive symptoms, within a routine care setting.

**Method:**

Secondary analysis from a 12‐month pragmatic randomized‐controlled trial delivered within the Improving Access to Psychological Therapies (IAPT) programme in England. Participants in the intervention arm were included if they met criteria for reliable recovery from depression (PHQ‐9) and anxiety (GAD‐7) at post‐treatment assessment. Survival analysis was used to assess durability of treatment effects and determine predictors to relapse at 3‐, 6‐ and 9‐month follow‐up. Hazard ratios predicting time‐to‐relapse were estimated with semi‐parametric Cox proportional hazards model.

**Results:**

Of the 241 participants in the intervention arm, 89 participants met the criteria for reliable recovery from depression and anxiety at the post‐treatment assessment. Of these 89 eligible cases, 29.2% relapsed within the 9‐month period, with 70.8% remaining in remission at 9 months post‐treatment. Of those who relapsed, 53.8% experienced a relapse of depression and anxiety; 7.7% experienced a relapse of depression only; and 38.4% experienced a relapse of anxiety only. Younger age, having a long‐term condition, and residual symptoms of anxiety at end‐of‐treatment were all significant predictors of relapse.

**Conclusions:**

This study is the first to explore the remission and relapse rates after an acute phase of iCBT treatment, within a routine, stepped‐care setting. The results add to the scarce literature on the durability of the effects of iCBT treatment in routine care settings, where patients are not typically followed up after receiving a completed course of treatment.

**Key Practitioner Message**

- iCBT has durable effects after an acute phase of treatment within a routine care setting.
- Relapse rates observed after iCBT treatment are comparable to findings observed in face‐to‐face psychotherapy.
- Closer follow‐up of younger users, those with long‐term conditions, and residual symptoms may be warranted
- Usage of iCBT continues beyond the acute treatment period, which may facilitate maintenance of effects.
- The content and delivery format of iCBT interventions make them a promising solution to maintenance of effects and long‐term remission after an acute phase of treatment.

## 1 INTRODUCTION

Depression and anxiety are leading causes of disability worldwide (World Health Statistics, 2017). The high rates of relapse and recurrence that characterize these mental health disorders are key factors that contribute to their burden. Between 39% to 56% of individuals who have recovered from their first episode will have one or more further episodes in their lifetime, and the risk of recurrence increases incrementally as a function of the number of episodes previously experienced (Bruce et al., 2005; Burcusa & Iacono, 2007; Vervliet et al., 2013). Given how common and consequential relapses can be, the importance of establishing the durability of treatment effects for anxiety and depression, and identifying factors that confer risk for relapse is readily apparent.

Individuals who complete cognitive behavioural therapy (CBT), the leading evidence‐based psychological treatment for depression and anxiety, tend to have lower rates of relapse compared to those who discontinue pharmacological treatment (Cuijpers et al., 2013; Hollon et al., 2006; Vittengl et al., 2007). Despite this, considerable risk of relapse also persists after CBT. For instance, recent meta‐analyses indicate that relapse rates range from 18.5% to 46.5% for depression (Wojnarowski et al., 2019) and from 13% and 42% for anxiety (Lorimer et al., 2021), when assessed over the course of 2 years following CBT treatment. Research examining factors that predispose risk for relapse following CBT is still relatively sparse. But, the broader literature on the clinical course of depression and anxiety has identified a range of potential predictors. Gender, age, employment status and socio‐economic background have all been implicated, but the persistence of residual symptoms and a higher number of previous episodes stand out as factors most consistently linked to increased likelihood of relapse in the long‐term (Lorimer et al., 2021; Wojnarowski et al., 2019).

In recent years, internet‐delivered CBT (iCBT) has been recognized as a viable alternative to traditional face‐face CBT, and it holds particular promise for its capacity to help address longstanding and pervasive issues pertaining to provider shortages and access problems (Mohr et al., 2021). Several studies have demonstrated both the effectiveness of iCBT in treating depression and anxiety (e.g., Andrews et al., 2018; Richards et al., 2020; Wright et al., 2019), and its capacity to produce remission rates that are comparable to face‐to‐face delivered CBT treatments (Andersson et al., 2018; Andrews et al., 2018; Karyotaki et al., 2018). However, very little is known to date about relapse rates following a course of iCBT. While meta‐analyses have shown that there is indeed a maintenance of effects for up to 18 months after iCBT treatment (Andersson et al., 2018; Andrews et al., 2018), these have only explored the durability of effects at an aggregated group‐level, and as such, they have not provided any information on relapse rates at an individual‐level. Two studies have investigated relapse rates after acute‐phase treatment for low‐intensity interventions, including but not limited to iCBT, in the Improving Access to Psychological Treatments (IAPT) stepped‐care model in the United Kingdom. Ali and colleagues found that 53% of cases relapsed within 1 year, with the majority occurring during the first 6 months (Ali et al., 2017). These figures are very similar to the other study that showed relapse rates of up to 65.8% at 24 months (52.8% at 12 months) for low‐intensity interventions in the same setting (Delgadillo et al., 2018). Another study, by Klein et al. (2018), examined the potential for internet‐based cognitive therapy to prevent relapse in a population of remitted recurrently depressed individuals. They did not find significant differences in relapse rates between the intervention and control groups over 24 months, with relapse rates of 43% and 49% in each group, respectively (Klein et al., 2018). Collectively, these studies have generally observed relapse rates that are higher than those observed in traditional face‐to‐face delivered CBT (Hollon et al., 2005; Scott et al., 2003). To the best of our knowledge, only two studies specific to iCBT for depression have published on relapse rates. Andersson et al. (2013) found relapse rates of 32.3% over the course of 42 months, and Schure et al. (2020) reported rates of 14.2% over the course of 12 months, respectively. However, these rates were derived from small samples (*n* = 34 and *n* = 28, respectively), and as such, there may be basis for questioning the generalizability of these findings. Andersson et al. (2013) also highlighted that a large proportion of participants (55%) had sought and received additional treatments in the follow‐up period, which further limits the conclusions that can be drawn about relapse rates and the extent to which patients maintain treatment gains following an acute phase of iCBT.

Taken together, the empirical literature exploring relapse rates following an acute phase of iCBT treatment for anxiety and depression is scarce, and our understanding of the factors associated with relapse in remitted patients is comparably limited. Therefore, the aim of the present study was to add to the existing literature by investigating relapse and remission rates following the completion of iCBT interventions for anxiety and depressive symptoms, within a routine care setting. More specifically, the objectives of this study were to quantify post‐treatment relapse rates at 3‐, 6‐ and 9‐month follow‐up from end of treatment, and to explore predictors of time‐to‐relapse.

## 2 METHOD

### 2.1 Ethics statement

This study utilized existing data from a trial that was originally approved by the NHS England Research Ethics Committee [REC Reference: 17/NW/0311].

### 2.2 Design

This is a secondary analysis of data from a recent pragmatic randomized controlled trial of internet‐delivered CBT interventions (iCBT) for depression and anxiety in routine care, with 12‐month follow‐up (Richards et al., 2020). In the main study, 361 participants were randomly allocated in a 2:1 ratio to the intervention (*n* = 241) or the waitlist control group (*n* = 120). For this secondary analysis, we looked at those in the intervention arm using the 3‐month point in the original study as baseline (which is the point where all participants in the intervention arm had completed their treatment), and follow‐up for 3–6 and 9 months thereafter.

### 2.3 Setting

The main trial was delivered within a stepped‐care model, the Improving Access to Psychological Therapies (IAPT) programme, within the National Health Service (NHS) in England. iCBT in this setting is offered as one of several low‐intensity interventions at Step 2 of the model, for individuals experiencing mild to moderate symptoms of depression and anxiety. It is supported by trained psychological wellbeing practitioners (PWPs), who deliver feedback on the users' progress through the content of the iCBT intervention via weekly asynchronous messages on the platform and on occasion complemented with telephone calls as required.

### 2.4 Intervention

The iCBT programs used in the current study were SilverCloud Health's ‘*Space from Depression*’ and ‘*Space from Anxiety*’ programs. SilverCloud Health is a provider of computerized psychological interventions for a variety of mental health and well‐being issues. The iCBT programs aim to develop and increase an understanding of one's thoughts, feelings and behaviours while applying a range of coping strategies such as mindfulness, behavioural activation, graded exposure, problem solving, activity scheduling and cognitive restructuring. They are delivered online through a variety of multimedia and interactive tools. Further details on the therapeutic components and content of each of the programs can be found in the protocol of the original RCT (Richards et al., 2018).

During their iCBT treatment, participants are supported by a psychological wellbeing practitioner (PWP) trained in the delivery of low‐intensity cognitive behaviour therapy interventions (Clark, 2011, 2018). The PWP introduces participants to their programme, monitors their progress throughout the trial and provides regular online and telephone reviews to participants, giving them feedback, guidance and responding to the work they complete between each review. Beyond a basic amount of participant information available to PWPs on the SilverCloud Health platform, participants can determine how much they want to share with their supporters. Over the intervention period, PWPs are advised to complete six to eight separate online reviews with participants, each taking approximately 10–15 min to complete. Supporters are supervised by case managers, responsible for monitoring the content of PWPs' online reviews and the corresponding CBT module and ensuring best practice standards of care.

### 2.5 Participants

Consistent with the healthcare service delivering the treatment, inclusion criteria for the overarching trial were (i) being aged between 18–80 years; (ii) presence of symptoms of depression and/or anxiety above clinical thresholds at initial assessment, as measured by the Patient‐Health Questionnaire‐9 (PHQ‐9; score ≥10) and/or Generalised Anxiety Disorder‐7 (GAD‐7; score ≥8); (iii) being deemed suitable for iCBT (i.e., internet access). Participants were excluded from the trial if they reported suicidal ideation or intent (PHQ‐9 question 9 score >2 and/or communicated at the time of clinical interview); psychosis; an organic mental health disorder; alcohol and/or drug misuse; or were current recipients of psychological treatment.

Given that the waitlist control group proceeded to receive treatment immediately after 8 weeks, the time period allocated to intervention, the acquisition of follow‐up data was limited to the active intervention arm. Accordingly, participants were included in this secondary analysis if they were allocated to the intervention arm and demonstrated a reliable improvement of depression or anxiety symptoms at the end of treatment, thus meeting criteria for reliable recovery (defined in detail below).

### 2.6 Measures and data sources

Two validated patient reported outcome measures are routinely used in IAPT services to monitor depression and anxiety symptoms: The PHQ‐9 is a self‐report measure of depression that has been widely used in research and is a regular screening measure in primary care settings (Spitzer et al., 1999). Each item is rated on 0 to 3 scale, yielding a summary score ranging from 0 to 27, where larger scores reflect a greater severity of depressive symptoms. A cut‐off ≥10 is used to detect clinically significant depression symptoms (Kroenke et al., 2001) and a reliable change index of ≥6 points is recommended to monitor improvement or deterioration over time (McMillan et al., 2010). The GAD‐7 comprises of 7 items measuring symptoms and severity of anxiety based on the DSM‐IV diagnostic criteria for GAD. Each item is also rated on a 0–3 scale, yielding a total anxiety severity score between 0 and 21. A cut‐off score ≥8 is recommended to identify the likely presence of a diagnosable anxiety disorder (Kroenke et al., 2007) with a reliable change index of ≥4 points (National Health Service (NHS), 2018a, 2018b; Richards & Borglin, 2011). Participants were sent reminders via email to complete these questionnaires on the SilverCloud platform at each follow‐up time point. If the questionnaires were not completed following two reminder emails, a member of the research team would call the participants and offer them the opportunity to complete the questionnaires over the phone. A small number of participants availed of the option to complete hardcopy versions of the questionnaires and post them to the research team.

### 2.7 Statistical analysis

Within IAPT, the definition for reliable recovery is a score that moves from caseness to non‐caseness (i.e., moves from above to below clinical cut‐offs), and exceeds the measurement error of that questionnaire (IAPT, 2014). A participant was considered to have met the criteria for reliable recovery if they (1) completed treatment with a score of ≤9 on the PHQ‐9 and ≤7 on the GAD‐7 and (2) demonstrated a reliable improvement of ≥6 points on the PHQ‐9 or ≥4 points on the GAD‐7, from baseline to end‐of‐treatment (i.e., 3 months). A participant was considered to have relapsed if (1) their post‐treatment score at 3‐, 6‐ or 9‐month follow‐up was ≥10 on the PHQ‐9 or ≥8 on the GAD‐7 and (2) they showed a reliable deterioration of ≥6 points on the PHQ‐9 questionnaire or ≥4 points on the GAD‐7, from the time of end‐of‐treatment to 3‐, 6‐, or 9‐month follow‐up. Participants were therefore categorized as in remission or relapsed at each time point. Therefore, participants who did not experience a relapse event by 9‐month follow‐up were considered to still be in remission.

Baseline demographic and clinical characteristics were described for the total sample making up our secondary analysis, and comparison between relapse and remission groups at baseline was done using chi‐square and *t* tests as appropriate. Usage data, including number of logins and time spent on the platform, were collected for all participants and compared between the relapse and remission groups using independent sample *t* tests.

A survival analysis was used to assess the durability of iCBT treatment effects over 9 months following treatment completion, and to determine predictors to relapse. Time‐to‐relapse, measured in months, was assessed using non‐parametric Kaplan–Meier (KM) curves (Kaplan & Meier, 1958). Kaplan–Meier curves plot the probability of survival (i.e., remission) over time. Hazard ratios predicting time‐to‐relapse were also estimated with a semi‐parametric Cox proportional hazards model (Cox, 1972). This model makes no assumptions about the shape of the baseline hazard function however, there are assumptions such as (i) independence of survival times, (ii) a change in predictors produce proportional changes in the hazard regardless of time and (iii) a linear association between the natural algorithm of the relative hazard and the predictors.

For these analyses, missing data were accounted for through censoring. Data points for each participant are censored when their survival time (i.e., time to relapse, in this study) cannot be accurately determined at any given time point. This happens for a number of reasons including (1) when data are not available for a participant at a given follow‐up time point, (2) when a participant drops out of the study and (3) when a participant does not experience the event, that is, does not relapse (or the study ends before they do). Therefore, each estimate at each time point is based solely on the observed data.

## 3 RESULTS

### 3.1 Sample characteristics

Of the 241 eligible participants in the main RCT, 89 met the eligibility criteria for inclusion in this secondary analysis. The mean age in the sample was 34.83 (SD = 13.39; range = 18 to 74); 77.5% (*n* = 69) were females; 81.8% (*n* = 72) were from a White British background; and 18.2% (*n* = 16) were identified as having a long‐term health condition. At the time of treatment completion, 76.5% (*n* = 65) were employed either full‐time or part‐time; 27.4% (*n* = 23) were taking medication; and 25% (*n* = 22) had a formal diagnosis of depression and/or anxiety disorder based on the Mini International Neuropsychiatric Interview 7.0.2 (MINI). Table 1 presents an overview of the PHQ‐9 and GAD‐7 scores at each time point for all 241 participants in the intervention arm, and the subset of 89 participants who met the criteria for this secondary analysis.

**TABLE 1:** PHQ‐9 and GAD‐7 scores for all participants in the intervention arm, and a breakdown of the scores for the ineligible and eligible subgroups

	Baseline	End‐of‐treatment	3 months	6 months	9 months
**Intervention‐arm, *n***	241	186	182	177	173
PHQ‐9 mean (SD)	14.4 (4.9)	8.2 (5.7)	7.2 (5.8)	6.8 (5.7)	6.8 (5.5)
GAD‐7 mean (SD)	12.7 (4.7)	7.4 (4.7)	6.9 (5.3)	6.5 (5.5)	6.1 (4.8)
**Ineligible, *n***	152	97	96	93	93
PHQ‐9 mean (SD)	15.2 (5.0)	11.9 (5.1)	9.2 (6.1)	8.9 (5.6)	8.8 (5.7)
GAD‐7 mean (SD)	13.3 (4.7)	11.2 (4.4)	9.1 (5.9)	8.5 (5.0)	7.4 (4.9)
**Eligible, *n***	89	89	86	84	80
PHQ9 mean (SD)	13.0(4.5)	4.0 (2.6)	4.9 (4.6)	4.6 (4.9)	4.5 (4.3)
GAD7 mean (SD)	11.5 (4.5)	3.2 (2.2)	4.5 (3.9)	4.2 (4.3)	4.5 (4.3)

In terms of residual symptoms at the end of treatment, 41.6% (*n* = 37) showed sub‐threshold symptoms of depression and 33.7% (*n* = 30) showed sub‐threshold symptoms of anxiety. Descriptive statistics and results from *t* tests and chi‐square tests comparing participants who did (*n* = 26, 29.2%), and did not (*n* = 63, 70.8%) experience a relapse event at any point during follow‐up are presented in Table 2.

**TABLE 2:** Descriptive statistics for included participants at 3 months (end‐of‐treatment)

	Remission (*n* = 63) *n* (%)	Relapse (*n* = 26) *n* (%)	Total (*n* = 89) *n* (%)	*Χ* ^2^	df	*p*
Age M (SD)	37.02 (13.79)	29.54 (10.88)	34.83 (13.39)	2.464^d^	87	0.016
Gender						
Male	17 (27)	3 (11.5)	20 (22.5)	2.520	1	0.112
Female	46 (73)	23 (88.5)	69 (77.5)			
Ethnicity^a^						
White	53 (85.5)	19 (73.1)	72 (81.8)	1.896	1	0.169
Other	9 (14.5)	7 (26.9)	16 (18.2)			
Long‐term condition						
Yes	10 (15.9)	6 (24)	16 (18.2)	0.795	1	0.373
No	53 (84.1)	19 (76)	72 (81.8)			
Employment status^b^						
Employed	47 (79.7)	18 (69.2)	65 (76.5)	1.091	1	0.296
Unemployed/student	12 (20.3)	8 (30.8)	20 (23.5)			
Medication^c^						
Taking	16 (27.6)	7 (26.9)	23 (27.4)	0.004	1	0.950
Not taking	42 (72.4)	19 (73.1)	61 (72.6)			
MINI diagnosis^a^						
Yes	15 (24.2)	7 (26.9)	22 (25)	0.073	1	0.787
No	47 (75.8)	19 (73.1)	66 (75)			
PHQ‐9 residual symptoms^e^						
Yes	24 (38.1)	13 (50)	37 (41.6)	1.074	1	0.300
No	39 (61.9)	13 (50)	52 (58.4)			
GAD‐7 residual symptoms						
Yes	20 (31.7)	10 (38.5)	30 (33.7)	0.371	1	0.542
No	43 (68.3)	16 (61.5)	59 (66.3)			

^a^ Total *n* = 88.

^b^ Total *n* = 85.

^c^ Total *n* = 84.

^d^ *T* test value.

^e^ Residual symptoms = PHQ‐9 score 5–9; GAD‐7 score 5–7.

### 3.2 Time‐to‐event analysis

**FIGURE 1:**
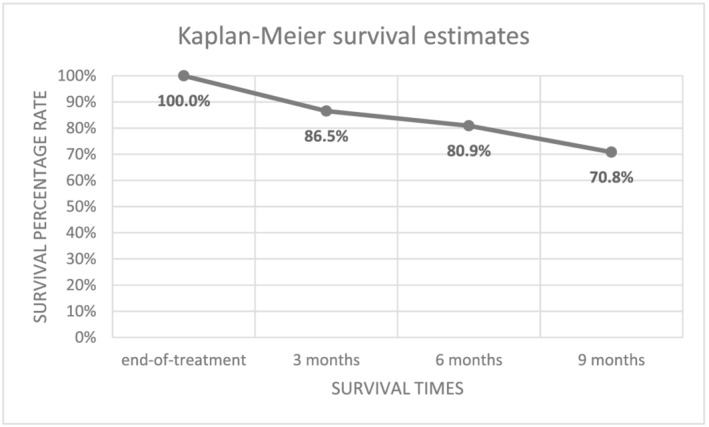
Survival analysis of remission and time‐to‐relapse following internet‐delivered cognitive‐behavioural therapy (iCBT). *Note*: *N* = 89 at end‐of‐treatment; 77 at 3 months; 72 at 6 months, 63 at 9 months

In total, after accounting for censored data points, 29.2% (*n* = 26) of cases were classified as relapse events within the 9‐month period. Therefore, 70.8% (*n* = 63) remained in remission at 9 months post‐treatment. Of those who relapsed, 53.8% (*n* = 14) experienced a relapse of depression and anxiety; 7.7% (*n* = 2) experienced a relapse of depression only; and 38.4% (*n* = 10) experienced a relapse of anxiety only. Figure 1 displays Kaplan–Meier (KM) survival estimates, where the curve denotes the overall proportion of cases remaining in remission at each time‐point. Most relapse events occurred at 6‐month follow‐up with the least number of cases observed at 9 months. Specifically, 46.2% (*n* = 12) of relapse events occurred within 3 months, 19.2% (*n* = 5) within 6 months and 34.6% (*n* = 9) within 9 months.

For further information about the proportions of participants who continued to show reductions, no change, and an increase in symptoms relative to the ‘end‐of‐treatment’ at each time point, please see Table S1.

### 3.3 Predicting relapse

Table 3 shows the results of the Cox proportional hazards model predicting time‐to‐relapse using multiple variables that were hypothesized a priori as potential prognostic factors. This model shows that age, long‐term condition and residual symptoms of anxiety at end‐of‐treatment were all significant predictors of relapse. Specifically, as age increases, the risk of a relapse event decreases (hazard ratio = 0.94, *p* = 0.01). In terms of long‐term condition, participants with a long‐term condition were over three times more likely (hazard ratios = 3.936, *p* = 0.02) to experience a relapse event within 9 months. Lastly, participants with residual symptoms of anxiety (GAD‐7 score of between 5 and 7) at end‐of‐treatment were over two times more likely (hazard ratio = 2.94, *p* = 0.046) to experience a relapse event than those with no or only minimal symptoms.

**TABLE 3:** Cox proportional hazard model predicting time‐to‐relapse

	Hazard ratio	SE	*Z*	*P*	95% CI
**End‐of‐treatment**					
Age	0.940	0.024	−0.258	0.008	0.898 to 0.984
Gender	2.981	0.779	1.402	0.161	0.648 to 13.724
Ethnicity	2.009	0.645	1.082	0.279	0.568 to 7.106
Long‐term condition	3.936	0.606	2.261	0.024	1.201 to 12.903
Employment status	1.680	0.590	0.880	0.379	0.529 to 5.430
MINI dep and/or anx diagnosis	0.758	0.613	0.454	0.651	0.228 to 2.519
Residual symptoms (PHQ‐9)	0.814	0.579	−0.356	0.772	0.262 to 2.530
Residual symptoms (GAD‐7)	2.937	0.865	2.258	0.046	1.019 to 8.466
**Baseline to end‐of‐treatment change**					
Medication (reference never took medication)					
Always on medication	1.904	0.614	1.049	0.295	0.571 to 6.347
Started taking medication.	0.412	1.193	−0.744	0.457	0.040 to 4.266
Stopped taking medication.	1.800	0.685	0.858	0.391	0.470 to 6.888
**Usage (baseline to end‐of‐treatment)**					
Number of logins	0.946	0.026	−0.154	0.878	0.946 to 1.049
Length of platform use	0.997	0.001	−1.000	0.573	0.997 to 1.002
Number of reviews	0.679	0.176	−0.239	0.811	0.679 to 1.353
**Usage (end‐of‐treatment to 9 months post‐treatment)**					
Number of logins	1.065	0.097	0.649	0.515	0.881 to 1.288
Length of platform use	0.966	0.006	−0.600	0.519	0.985 to 1.008

## 4 DISCUSSION

This study is the first to explore the remission and relapse rates after an acute phase of treatment with iCBT only, within a routine, stepped‐care setting and over a period of 9 months post‐treatment. The results add to the scarce literature on the durability of the effects after iCBT treatment in routine care settings, where patients are not typically followed up after receiving a completed course of treatment. Our main findings showed that 70.8% of patients who attained reliable recovery after iCBT treatment remained in remission after 9 months. Younger age, having a comorbid long‐term condition, and reporting residual anxiety symptoms at the end of treatment were all indicative of a significantly higher risk of relapse within the follow‐up period.

In our study, 29.2% of patients relapsed over a period of 9 months post‐treatment. The relapse rates observed here are lower than those reported by Klein et al. (2018) and other studies examining low‐intensity interventions including but not limited to iCBT (Ali et al., 2017; Delgadillo et al., 2018). There are several methodological differences across these studies that limit direct comparisons. However, it is important to point out that Klein and colleagues specifically recruited a sample of *recurrently* depressed participants. Thus, given that it is well established that the risk of recurrence increases as a function of the number of episodes previously experienced (Bruce et al., 2005; Burcusa & Iacono, 2007; Vervliet et al., 2013), it may not be too surprising that their sample of participants had higher rates of relapse. The discrepancies between the relapse rates we observed and those reported by both Ali et al. (2017) and Delgadillo et al. (2018), may possibly be at least partially attributable to the fact that these studies did not distinguish between guided self‐help (i.e., bibliotherapy) and iCBT. The literature on relapse rates for bibliotherapy is scarce, but data from two studies with relatively small sample sizes suggest relapse may indeed be higher in this type of therapy (Floyd et al., 2006; Smith et al., 1997). Our study looked exclusively at iCBT interventions that are well‐established and embedded in routine care pathways. Additionally, the iCBT programs evaluated in this study continued to be available to participants in an unsupported capacity beyond the end of the treatment period, allowing participants to log in and use the programme for up to a year. This advantage that iCBT has over other treatments, including face‐to‐face therapy, may have played a role in the maintenance of the effects over time.

Regarding predictors of relapse at follow up, we found that younger age was associated with higher risk of relapse, which aligns with research on traditional CBT (Gonzales et al., 1985), and some forms of low‐intensity CBT (Lorimer et al., 2021). This finding is also consistent with the more general observation that the frequency of episodes of depression and anxiety reduce with age (Asselmann et al., 2019;Blazer, 2010; Jorm, 2000), which in turn may be due to several factors such as age‐related reductions in emotional responsiveness, increased emotional control, and psychological immunnisation to stressful events (Blazer, 2010; Jorm, 2000). Residual anxiety symptoms were found to be a predictor of relapse, but these effects were not found for residual depressive symptoms. This was surprising, but given that our definition for relapse stated that relapse could be due to a significant increase in *either* depression or anxiety symptoms, it seems that residual symptoms of anxiety played a larger role in determining relapse in our sample. Our results align with previous research in traditional CBT and in low‐intensity interventions where residual symptoms have been consistently identified as predictors of relapse for depression and anxiety disorders (Ali et al., 2017; Lorimer et al., 2021; Wojnarowski et al., 2019). This study also found that the presence of long‐term conditions was a significant predictor of relapse at 12 months, which has not been reported in previous research examining predictors of relapse following iCBT, or traditional face‐to‐face CBT. The absence of evidence for long‐term conditions being linked to higher relapse rates to date is likely due to how most studies evaluating treatment outcomes for depression and anxiety impose criteria that exclude individuals with long‐term conditions in an effort to limit heterogeneity in their study samples. Accordingly, much more research on treatment outcomes and relapse rates for this cohort is warranted. Nonetheless, the increased risk of relapse for individuals with long‐term conditions that we observed is compatible with research which has established that comorbid physical and psychological conditions are associated with poorer patient outcomes than either condition alone (Aquin et al., 2017; Stein et al., 2006).

Given that this study was conducted within a naturalistic setting, it offers valuable insights on how services offering iCBT, including but not limited to IAPT, may be able to tailor their care pathways to improve outcomes for patients. For instance, the findings from this study suggest that young people, individuals with residual anxiety symptoms at the end of treatment, and individuals with long‐term conditions are more susceptible to relapse following an acute phase of iCBT for depression and anxiety. Services could use this information to inform them about who might need to be prioritized for the provision of targeted follow‐up support which may prevent relapse from occurring. Overall, one could assume that this would help alleviate future costs and improve resource allocation within services in the long‐term.

The effectiveness of psychological interventions for relapse prevention has been demonstrated for both depression (Piet & Hougaard, 2011; Vittengl et al., 2007) and anxiety, though for anxiety, this has been limited mostly to panic disorder (van Dis et al., 2020). Despite the evidence base underpinning this, the implementation of relapse prevention programs into routine care services is often hindered due to lack of resources. The flexibility of iCBT and the fact that it requires a low level of support makes it a suitable alternative to be offered as a relapse prevention intervention within clinical services. This would be especially relevant during the first 6 months after discontinuation of treatment, when most relapses occur (Ali et al., 2017; Klein et al., 2018). In one study, an iCBT programme for relapse prevention was offered to partially remitted patients and the protective effects were observed up to 2 years later (Holländare et al., 2013). Therefore, the utilization of iCBT for relapse prevention holds promise for reducing relapse rates in individuals who respond to an acute phase of treatment.

Our study is not without limitations. We did not have a control group at follow up, which prevents us attributing the observed maintenance of the effects solely to the iCBT intervention. However, reductions of symptoms during follow‐up have been shown in previous iCBT studies (Andersson et al., 2018; Andrews et al., 2018). The design of the study allowed us to follow up patients up to 9 months post‐treatment, which prevents us from comparing results at a later time, when relapse rates might be higher. Future studies should include longer follow ups to explore the durability of the effects up to 2 years. Finally, the measurements used to calculate relapse are self‐reported and no objective clinical assessment was completed with participants beyond 3 months. However, routine outcome assessment using the PHQ‐9 and GAD‐7 is common in these service settings, and this study provides further support for how using them to continue to acquire information about patients' symptoms beyond the end of treatment can provide an important means for rapidly identifying both remitters and relapsers.

## 5 CONCLUSIONS

The ability to identify patients at risk of relapse is an important challenge, as recurrence of depression and anxiety may require further treatment, which can lead to higher personal, economic and societal costs. This paper adds to the literature on the durable effects of iCBT and factors that may predict risk of relapse after an acute phase of iCBT treatment. Results showed that during a 9‐month period after iCBT treatment, the relapse rates were comparable to face‐to‐face CBT. Our findings on predictors suggest that remitters who have residual symptoms of anxiety at post‐treatment, who are young and suffer from long‐term conditions have a higher risk for relapse. Early identification of these individuals and the provision of supplementary iCBT sessions or other targeted relapse prevention support could help to improve the likelihood that these individuals stay in remission after successful treatment. The routine care setting for this trial supports the ecological validity to the results as well as the potential to modify care pathways to leverage iCBT in relapse prevention.

## CONFLICT OF INTEREST

Authors Palacios, Enrique, Mooney, Farrell, Earley, Duffy, Eilert, Harty and Richards are employees of SilverCloud Health and Timulak serves as a research consultant for SilverCloud Health. SilverCloud Health is a commercial entity that develops computerized psychological interventions for depression, anxiety, stress and comorbid long‐term conditions and sells these to Health Services globally. In England, the SilverCloud service is delivered free to patients through the Improving Access to Psychological Therapies (IAPT) programme. Commercial departments within SilverCloud Health played no role in the analysis or interpretation of data.

## Supporting information


**Table S1.** Proportions of participants who showed reductions, no change, and an increase in symptoms relative to the ‘end‐of‐treatment’ at each time‐point

## Data Availability

Data supporting these findings, along with all coding and modelling done as part of the statistical analysis, are available on request from the corresponding author.
