# Supplementary material for: Durability of treatment effects following internet‐delivered cognitive behavioural therapy for depression and anxiety delivered within a routine care setting
Source: Clin Psychol Psychother. 2022 Apr 26;29(5):1768–77. doi: 10.1002/cpp.2743 (PMC9790710; doi:10.1002/cpp.2743)
Supplement: Supplementary file 1 — Table S1. Proportions of participants who showed reductions, no change, and an increase in symptoms relative to the ‘end‐of‐treatment’ at each time‐point [file CPP-29-1768-s001.docx]

**Durability of treatment effects following internet-delivered cognitive behavioral therapy for depression and anxiety delivered within a routine care setting**

Jorge Palacios^a,b^, Angel Enrique^a,b^, Olwyn Mooney^a^, Simon Farrell^a^, Caroline Earley^a^, Daniel Duffy^a^, Nora Eilert^a,b^, Siobhan Harty^a^, Ladislav Timulak^b^, Derek Richards^a,b^

^a^SilverCloud Research, SilverCloud Health, One Stephen Street Upper, Dublin, Ireland.

^b^E-mental Health Research Group, School of Psychology, Aras an Phiarsaigh, Trinity College Dublin, Ireland.

**Supplementary Material**

**Table S1.** Proportions of participants who showed reductions, no change, and

an increase in symptoms relative to the ‘end-of-treatment’ at each time-point

| **Change relative to end-of-treatment** | **3-months** | **6-months** | **9-months** |
| --- | --- | --- | --- |
| **Reduction in symptoms** |  |  |  |
| PHQ9 | 37.1% | 40.5% | 37.1% |
| GAD7 | 32.6% | 39.3% | 30.3% |
| **No change** |  |  |  |
| PHQ9 | 11.2% | 10.2% | 14.6% |
| GAD7 | 14.6% | 14.6% | 16.9% |
| **Increase in symptoms** |  |  |  |
| PHQ9 | 48.3% | 43.8% | 38.2% |
| GAD7 | 49.4% | 39.3% | 42.7% |
| **Missing Data** |  |  |  |
| PHQ9 | 3.4% | 5.6% | 10.1% |
| GAD7 | 3.4% | 6.7% | 10.1% |
